# *CsIAGLU* Regulates the Angle of Leaf Petiole by Affecting Endogenous Content of Auxin in Cucumber (*Cucumis sativus* L.)

**DOI:** 10.3390/genes13122216

**Published:** 2022-11-25

**Authors:** Jiacai Chen, Yuxiang Huang, Xiaofeng Liu, Guangxin Chen, Liu Liu, Zhihua Cheng, Weiyuan Song, Lijie Han, Shaoyun Wang, Liming Wang, Min Li, Xiaolan Zhang, Jianyu Zhao

**Affiliations:** State Key Laboratories of Agrobiotechnology, Joint International Research Laboratory of Crop Molecular Breeding, Beijing Key Laboratory of Growth and Developmental Regulation for Protected Vegetable Crops, Department of Vegetable Sciences, China Agricultural University, Beijing 100193, China

**Keywords:** *CsIAGLU*, leaf petiole angle (LPA), auxin (IAA), cucumber, auxin glycosylation

## Abstract

The leaf angle is an important factor determining plant shoot architecture that may boost crop yield by increasing photosynthetic efficiency and facilitating high-density planting. Auxin is an important phytohormone involved in leaf angle regulation. Here, we identified two Single-Nucleotide Polymorphisms (SNPs) in the Indoleacetic Acid (IAA) glucosyltransferase gene *CsIAGLU* in 80 re-sequenced cucumber lines, of which the *CsIAGLU^717G,1234T^* is the dominant allele associated with a small leaf pedicle angle (LPA), whereas *CsIAGLU^717C,1234A^* is linked with a large LPA. *CsIAGLU* was highly expressed in leaves and petioles. In natural cucumber populations, the expression of *CsIAGLU* was negatively correlated with the LPA. The mutation of *CsIAGLU* induced by the CRISPR-Cas9 system resulted in elevated free IAA levels and enlarged cell expansion on the adaxial side of the petiole base, thus producing a greater LPA. Consistently, exogenous IAA treatment led to increased LPA and cell size. Therefore, our findings suggest that *CsIAGLU* functions as a negative regulator of LPA development via auxin-mediated cell expansion in cucumber, providing a valuable strategy for cucumber breeding with small LPAs.

## 1. Introduction

Increasing world population and declining arable land mean that there is an ever increasing need for elevated crop yield per unit area. Plant architecture is a comprehensive agronomic trait specifying crop yield when grown in high-density conditions that is composed of multiple factors, such as plant height and branching. The leaf angle is an important shoot architecture trait that may boost crop yield by increasing photosynthetic efficiency and facilitating high-density planting [1,2,3,4]. Therefore, the leaf angle has been selected as a target trait during crop breeding of ideal shoot architecture [5]. The leaf angle refers to the inclination between the leaf and the stem. However, due to distinct anatomy, there are some differences in defining the leaf angle in monocots and eudicots. In monocotyledonous plants, such as rice and maize, the mature leaf consists of three parts: the leaf, the leaf sheath and the leaf tongue [6,7]. The leaf angle is defined as the inclination between the vertical stem and the midrib of a leaf blade [8]. In dicotyledonous plants, such as soybean and thale cress (*Arabidopsis thaliana*), the mature leaf is composed of the leaf and the petiole. The leaf angle refers to the angle between the main stem and the petiole, also known as leaf petiole angle (LPA). 

In monocots, the leaf angle is determined by the size of the cells of the collar tissue and is involved in multiple hormones [9,10,11]. Several studies have shown that brassinosteroid (BR) is a key factor in regulating the leaf angle. The loss of function of *OsDWARF2*, *OsDWARF4* and *OsDWARF11* mutants was reported to decrease the endogenous level of BR and lead to the erect leaf phenotype [12,13,14,15,16]. The work of Sun et al. showed that the BR signal inhibits abaxial sclerenchyma cell proliferation by coordinately regulating *CYCU4;1* through BES1 (BR-SIGNALING KINASE1) and GSK3 (Glycogen synthase kinase-3) kinases, thereby displaying a decreased leaf angle [17]. *BRASSINAZOLE RESITANT1* (*BZR1*), the negative regulator of the BR signaling pathway, positively regulates the leaf angle in rice, and the knock down of *BZR1* results in a reduced leaf angle [18,19]. Similarly, the B3-domain transcription factor *ZmRAVL1* (*Related to ABI3/VP1-Like 1*) participates in leaf angle regulation in maize by altering the endogenous BR content via *brd1* (*brassinosteroid C-6 oxidase 1*) [20]. Thus, BR biosynthesis and signal-transduction-related genes are extensively involved in modulating the leaf angle [8].

Moreover, BR generally crosslinks with other hormone signaling pathways in fulfilling its function in leaf angle regulation. *OsREM4.1* (*REMORIN GROUP 4, MEMBER 1*) is induced by ABA (abscisic acid) and inhibits BR signaling. The overexpression of *OsREM4.1* results in a small leaf angle, resembling the phenotype of BR-deficient mutants [21]. There are complex interactions between BR and GA (gibberellin) in regulating the leaf angle [22]. The loss of function of *OsSPY* (*OsSPINDLY*), the negative regulator of GA signaling, leads to an increased leaf angle by inhibiting the expression of BR biosynthetic genes *D11*, *D2*, *OsCPD1* and *OsDWARF* [23]. *OsGSR1* (*gibberellin-stimulated transcript*) is induced by GA and interacts with DIM/DWF1 to enhance BR biosynthesis and promote the leaf angle in rice [24]. In addition, BR can induce GA biosynthesis, and while GA is excessive, the synthesis of BR is also inhibited, forming a feedback loop to maintain the proper hormone levels and thus a normal leaf angle [25]. The exogenous application of GA3 can restore the upright leaf phenotype of BR mutants *na2-1* and *na1-1*, which originally have horizontal leaf angles [26]. Recent studies have shown that cytokinin is also involved in the regulation of the leaf angle in rice. The overexpression of cytokinin oxidase OsCKX3 (Cytokinin oxidase/dehydrogenase 3) increases the leaf angle, while the mutants display a smaller leaf angle. The expression of *CYCU4;1* decreases in *OsCKX3* overexpression lines, but increases in *Osckx3* mutants, suggesting that BR and cytokinin may act antagonistically to regulate the leaf angle through *CYCU4;1* [27]. 

Indoleacetic Acid (IAA) is another important hormone that seems to play important roles in regulating the leaf angle [22]. The decrease in the IAA content results in a larger leaf angle, while increased IAA causes a smaller leaf angle in maize [28]. The loss of function of *FISH BONE* (*FIB*; encodes a tryptophan aminotransfer) leads to reduced IAA content and a larger leaf angle [29]. IAA amino synthetase OsGH3.1 catalyzes the binding of IAA with various amino acids to maintain auxin homeostasis. Gain-of-function mutant *lc1-D* displays reduced content of free IAA, which stimulates cell elongation at the lamina joint and increases the leaf angle in rice [30]. In addition, the downregulated expression of auxin receptor genes *OsTIR1* and *OsAFB2* leads to an enlarged leaf angle [31], while the loss of function of auxin efflux carriers *ZmPGP1* [32] and *GmPIN1* [33] shows a reduced leaf angle. Thus, these studies indicate that IAA negatively regulates the leaf angle. However, other reports show that increased IAA leads to a greater leaf angle. While these studies indicate that higher IAA leads to a decreased leaf angle, other reports show that higher IAA results in an increased leaf angle [34,35,36]. In maize and sorghum, plants containing the functional genes *BRACHYTIC2* (*BR2*; maize) and *DWARF3* (*Dw3*; sorghum) have a larger leaf angle due to elevated free IAA levels [37,38]. In wheat, *TaHST1L* overexpression lines display an increased tiller angle and higher IAA content, while RNAi lines show a significantly smaller tiller angle and lower IAA levels [39]. It can be seen that the homeostasis of endogenous auxin is crucial for the development of the leaf angle.

The homeostasis of endogenous IAA is regulated by a series of processes, including biosynthesis, transportation, oxidation, hydrolysis and the formation of conjugates. As the major storage form of auxin, auxin conjugates play a key role in regulating the effectiveness of endogenous free IAA. In plants, about 75% of IAA is stored in the form of conjugates or catabolites, thus providing a powerful and rapid response system to fine-tune the level of IAA [40]. Studies on different plants showed that the most abundant reversible IAA inactive forms are conjugates of IAA with sugars (such as glucose) [41,42,43]. Sugar conjugation has higher stability and water solubility and has been considered as a biomarker mechanism to control the compartmentalization and metabolite activity [44]. UDP-glycosyltransferases (UGTs) catalyze the transfer of uridine diphosphate activated monosaccharides to a variety of compounds, including auxin. At present, through in vivo and in vitro experiments, it has been found that some UGTs play a role in the reversible conversion of IAA to IAA-glucose (IAA-glc). However, there are no reports on IAA glucosyltransferase in cucumber.

Cucumber is an important vegetable crop with a long history of worldwide cultivation. The leaf petiole angle (LPA) in cucumber may not only affect the crop yield and planting density, but also influence the fruit quality and disease incidence due to light capturing and air movement. In cucumber production practice, the horizontal LPA requires more frequent removal of old leaves, thus needing extra labor costs, than the erect LPA. Dissecting the mechanism of LPA regulation is of great significance to cucumber breeding for high yield, superior quality and effective production. However, no studies have been reported about cucumber LPA development yet. Here, we identified that an indole-3-acetic acid glucosyltransferase gene (*CsIAGLU*) negatively regulates the LPA by modulating the endogenous IAA level at the base of the petiole and the adaxial cell size in cucumber. 

## 2. Materials and Methods

### 2.1. Plant Materials and Growth Conditions

Cucumber inbred line XTMC was used for genetic transformation. Cucumber inbred lines 17s-97, 17s-104, 17s-133, 17s-134, 17s-204, 17s-205, 17s-207, 17s-209 and 17s-212 with different leaf petiole angles were used for expression analyses. Cucumber seeds germinated in the dark at 25 ℃ and then grew in the growth chamber under light conditions at 25 ℃ for 16 h and in the dark at 18 ℃ for 8 h until the three-true-leaf stage. Then, the seedlings were transferred to the greenhouse, with standard procedures for water and fertilizer management, and pest control.

### 2.2. Phylogenetic Analysis

In order to identify the homologous protein of CsIAGLU in cucumber, all amino acid sequences encoding the IAA glucosyltransfer gene in *Arabidopsis* were used as queries for BLASTp searches at NCBI. IAA glucosyltransferase sequences from cucumber, Arabidopsis, maize and rice were aligned using ClustalW in MEGA6.0 and used to generate a phylogenetic tree utilizing the neighbor-joining method with 1000 bootstrap replications [45]. Protein domains were analyzed using SMART (http://smart.embl-heidelberg.de/ (accessed on 16 August 2022)). The accession numbers of all sequences are provided in Table S1.

### 2.3. CRISPR/Cas9-Mediated Mutations in Cucumber

To generate mutations in *CsIAGLU* using the CRISPR/Cas9 system, specific target sites were obtained from the website (http://cbi.hzau.edu.cn/CRISPR2/ (accessed on 20 October 2018)). The corresponding guide RNAs were cloned into pKSE402G, which contained a green fluorescent protein (GFP) reporter [46,47]. Then, they were transferred into cucumber inbred line XTMC using the optimized cotyledon transformation method as described previously [48]. In order to identify the transgenic lines, total DNA was extracted from T1 transgenic plants, and specific primers were used to amplify the fragments of target sites and sequence them. The primer information is listed in Table S2.

### 2.4. RNA Extraction and Expression Analysis

According to the manufacturer’s instructions, total RNA was isolated using Eastep^®^ Super Total RNA Extraction Kit (Promega, Madison, WI, USA) and reverse-transcribed into cDNA using FastKing gDNA Dispelling RT SuperMix Kit (Tiangen, Beijing, China). RT-qPCR assays were performed with TB Green^®^ Premix Ex TaqTM II (Takara, Kyoto, Japan) using CFX384 Real-Time PCR System (BIO-RAD, Hercules, CA, USA). Three biological and three technical replicates were conducted for each gene. The cucumber *Ubiquitin* extension protein gene (CsaV3_5G031430) was used as an internal control.

In order to analyze the expression pattern of *CsIAGLU*, samples of female flower, stem, petiole base, petiole, leaf base and leaf were collected for total RNA extraction. For the expression analysis of *CsIAGLU* in different cucumber germplasms, the petiole base of the fourth leaf counting from the top was collected for total RNA extraction. The primer sequences are listed in Table S2.

### 2.5. RNA In Situ Hybridization

The cucumber shoot apexes of 21-day-old seedlings were fixed in 3.7% formal-acetic-alcohol solution and stored at 4 °C until use. In situ hybridization was performed as described previously [49,50]. The *CsIAGLU* probes were designed according to the specific region of the corresponding CDs. Sense and antisense probes were synthesized using PCR amplification and were in vitro-transcripted with DIG RNA Labeling Kit (Roche, Basel, Switzerland) using SP6 and T7 polymerases, respectively, following the manufacturer’s instructions. The primer sequences are listed in Table S2.

### 2.6. Extraction and Quantification of Endogenous Auxin

In order to measure the auxin (IAA) content in wild-type and transgenic plants, about 0.1 g fresh samples were collected from the petiole base of the third and fifth leaves counting from the top of cucumber plants for auxin extraction. Three biological repeats were conducted for each genotype. An enzyme-linked immunosorbent assay was used to extract, purify and quantify endogenous auxin according to a method described previously [51].

### 2.7. Histology Observation

The petiole bases of the largest leaves were used as samples and fixed with 3.7% (*v*/*v*) FAA solution. The subsequent dehydration, embedding, sectioning, dewaxing and rehydration of the samples were performed for in situ hybridization [49,50]. After rehydration, the samples were immersed in 0.5% toluidine blue and dyed for 30 min. Subsequently, excess toluidine blue was washed off with water. Sample observation and cell measurement were performed using an optical microscope (Olympus D72, Japan). Three biological repeats were conducted for each genotype. 

### 2.8. Haplotype Analysis

A total of 80 cucumber inbred lines were sequenced, and BWA (http://superb-sea2.dl.sourceforge.net/project/bio-bwa (accessed on 28 October 2022))) was used to compare reads into the cucumber reference genome to obtain the SAM files [52]. Then, samtools (https://github.com/samtools/samtools (accessed on 26 October 2022)) was utilized to convert the SAM files to BAM files, sort reads and remove duplicate reads from the BAM files. BCFtools was used for SNP calling (https://github.com/samtools/bcftools (accessed on 26 October 2022)) to obtain the information of mutation sites [53]. 

## 3. Results

### 3.1. Identification of CsIAGLU Associated with Leaf Petiole Angle in Cucumber

The leaf angle is one of the important characteristics of ideal plant architecture [5] that affect crop yield. The mature cucumber leaf is composed of petiole and leaf. Similar to soybean, the leaf angle of cucumber refers to the angle between the main stem and the petiole and is called leaf petiole angle (LPA). Based on the LPA of the largest leaf, cucumber leaves are divided into three groups: upright (<30°), semi upright (30°~60°) and flat (60°~90°).

**Figure 1:**
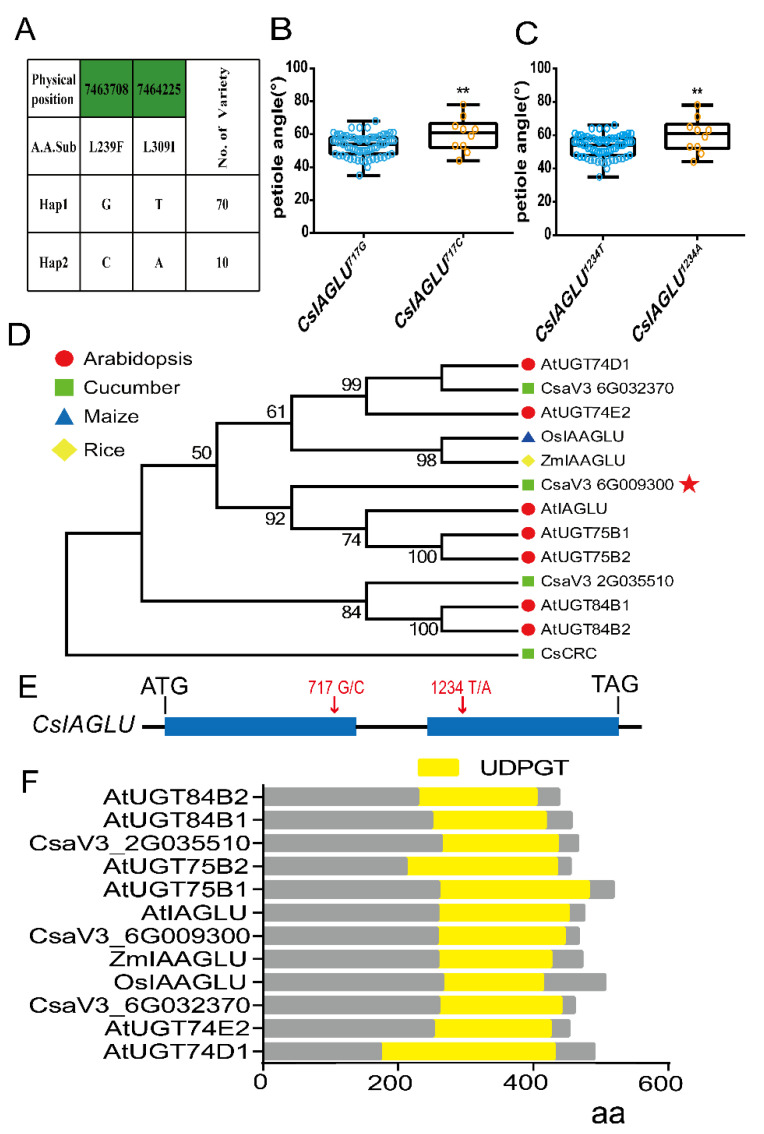
Identification of *CsIAGLU* in cucumber: (**A**) Two haplotypes of *CsIAGLU* are defined by the variants, and the accession numbers of each haplotype are labeled on the right. (**B**,**C**) Boxplots of distribution of LPAs in 80 cucumber lines containing different *CsIAGLU* haplotypes, *CsIAGLU^717G/C^* (**B**) and *CsIAGLU^1234^^T/A^* (**C**). Median values are indicated by horizontal lines within boxes, and the range of the 25% to 75% of the data is represented by the box height. A significance analysis was conducted with the two-tailed Student’s t-test (** *p* < 0.01). Values are means ± SDs. (**D**) Phylogenetic tree of the IAA glucosyltransferase proteins from thale cress, cucumber, rice and maize. The red star represents CsIAGLU in cucumber. (**E**) Schematic diagram of the gene structure of *CsIAGLU*. The black line represents introns, and the blue boxes represent exons. (**F**) Domain analysis of CsIAGLU homologous protein. The yellow boxes represent the UDPGT (UDP -gluconeosyl and UDP -glucosyltransfer) domain.

In order to explore the key regulator of the cucumber LPA, we analyzed the re-sequenced data of 80 cucumber inbred lines with varying LPAs. Among these lines, we identified a gene, *CsaV3_6G009300* (denoted hereafter as *CsIAGLU*), containing two SNP variations (717^G/C^ and 1234^T/A^) in the genomic sequence, that is closely correlated with the LPA in cucumber (Supplemental Data 1). Specifically, 717^G^ and 1234^T^ are the dominant alleles that are linked, whereas 717^C^ and 1234^A^ are the minor alleles, which are also closely linked (Figure 1A). The average LPA of the *CsIAGLU^717G^*^,*1234T*^ lines was 53.1 ± 6.3° (*n* = 70), which was significantly smaller than that of the *CsIAGLU^717C^*^,^*^1234A^* lines (59.8 ± 10.3°, *n* = 10) (Figure 1B,C), indicating that these two SNPs in *CsIAGLU* may have been selected in cucumber breeding with smaller LPAs. 

The phylogenetic analysis showed that CsIAGLU, AtIAGLU, AtUGT75B1 and AtUGT75B2 are clustered into the same clade (Figure 1D). The *CsIAGLU* gene contains two exons and one intron, encoding 467 amino acids (Figure 1E). Protein structure prediction indicated that CsIAGLU contains a conserved UDPGT (UDP -glucoronosyl and UDP -glucosyltransferase) domain similar to the homologous genes in thale cress, maize and rice (Figure 1F). The UDPGT domain has the activity of glucosyltransferase; thus, CsIAGLU is speculated to have the function of IAA glucosyltransferase in cucumber.

### 3.2. Expression Patterns of CsIAGLU in Cucumber

**Figure 2:**
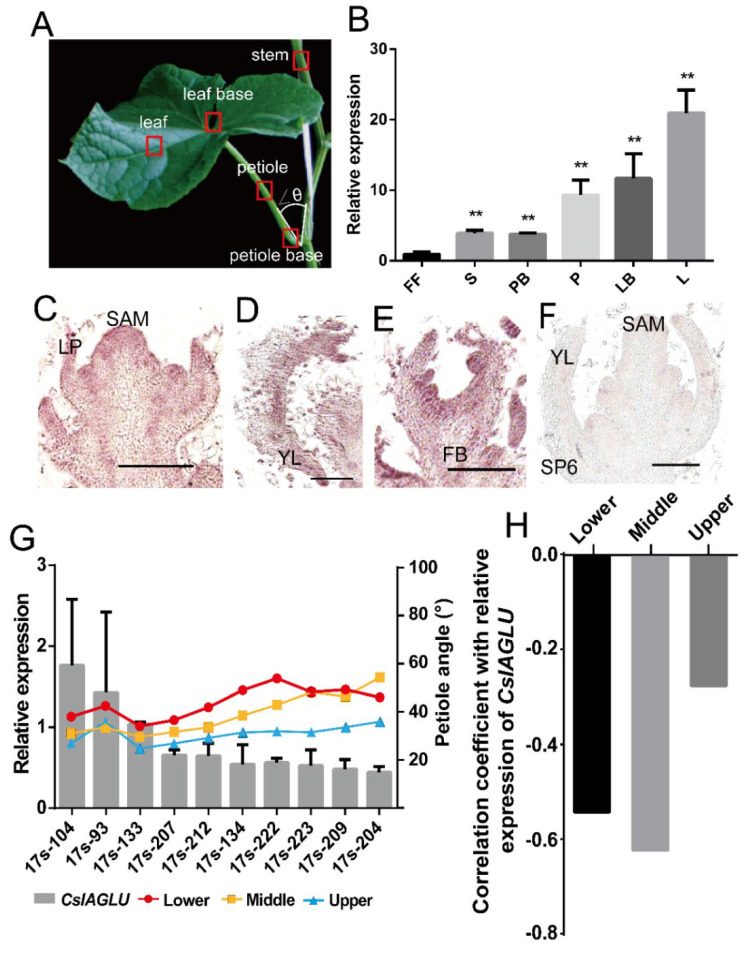
Correlation analysis of *CsIAGLU* expression and LPA in cucumber: (**A**) The image of a cucumber leaf. ∠θ refers to the LPA. The red boxes refer to tissue samples used for expression analysis in (**B**). (**B**) qRT-PCR analysis of *CsIAGLU* in different tissues. FF, female flower; S, stem; PB, petiole base; P, petiole; LB, leaf base; L, leaf. *CsIAGLU* transcripts were quantified using *CsUBI* as the internal standard. A significance analysis compared to FF was performed with the two-tailed Student’s *t*-test (** *p* < 0.01). Values are means ± SDs (*n* = 3). (**C**–**F**) In situ hybridization analysis of *CsIAGLU*: shoot apex (**C**), young leaf (**D**) and floral bud (**E**). The sense *CsIAGLU* probe was hybridized as a negative control (**F**). Scale bar, 1 μm. (**G**) Expression of *CsIAGLU* in different cucumber lines with various LPAs. The gray columns represent the expression of *CsIAGLU*, and the line charts represent the LPAs in lower, middle and upper parts. Values are means ± SDs (*n* = 3). (**H**) Correlation analysis between *CsIAGLU* expression and LPA.

In order to characterize the function of *CsIAGLU*, its expression pattern was analyzed. The qRT-PCR results of different cucumber tissues showed that the expression levels of *CsIAGLU* were highly enriched in the leaf, leaf base and petiole, while they were moderately expressed at the petiole base, stem and female flower (Figure 2A,B). In situ hybridization indicated that the *CsIAGLU* signal was observed in the shoot apical meristem (SAM), the adaxial side of the leaf primordium and adaxial floral organs (Figure 2C–E). No signals were detected following hybridization with the sense *CsIAGLU* probe (Figure 2F). To further explore the causal relationship between *CsIAGLU* and the cucumber LPA, the expression levels of *CsIAGLU* in ten cucumber germplasms with different LPAs were analyzed using qRT-PCR. Considering that the LPA at different nodes varies significantly in cucumber, we divided the plant into the lower, middle and upper parts and calculated the average LPA of each part. Among the germplasms, the 17s-104, 17s-93 and 17s-133 lines had the highest levels of *CslAGLU* and the lowest LPAs, while the 17s-223, 17s-209 and 17s-204 lines had the lowest levels of *CslAGLU* and the highest LPAs, demonstrating a negative correlation between *CsIAGLU* and the LPA (Figure 2G). The correlation coefficients between *CsIAGLU* expression and the LPA were −0.518, −0.627 and −0.255 for the lower, middle and upper parts, respectively (Figure 2H), suggesting that *CsIAGLU* may play an important role in LPA development in cucumber.

### 3.3. Mutation of CsIAGLU Alters Leaf Petiole Angle in Cucumber

**Figure 3:**
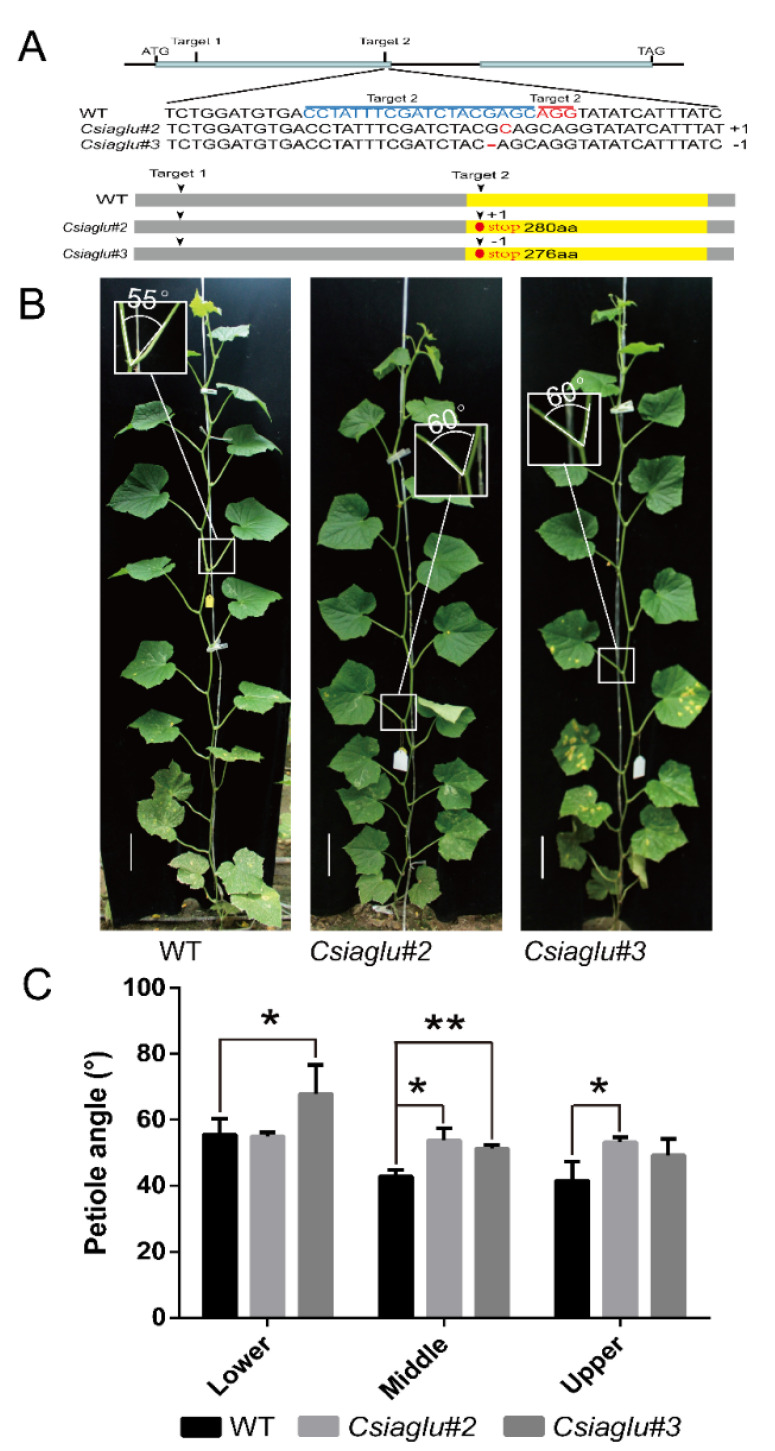
Phenotypic characterization of WT and *Csiaglu* mutant plants: (**A**) Identification of *Csiaglu* mutants. Two targets were selected at the first exon of *CsIAGLU*; the sgRNA targets are marked in blue, and protospacer-adjacent motif (PAM) sites are highlighted in red. The genotype analysis indicated that the *Csiaglu#2* allele inserted 1 bp at the second target and the *Csiaglu#3* allele deleted 1 bp at the second target. The red dots represent the termination codon, and the yellow boxes indicate the UDPGT domain. (**B**) Representative images of WT and *Csiaglu* mutant plants. The white boxes indicate the LPA of the largest leaf. Scale bars represent 10 cm. (**C**) Statistical analysis of LPAs of WT and *Csiaglu* mutants. Values are means of LPAs from the same part of 3 independent plants. A significance analysis was conducted with the two-tailed Student’s *t*-test (* *p* < 0.05 and ** *p* < 0.01). Values are means ± SDs.

To validate the function of *CsIAGLU* in the cucumber LPA, the CRISPR/Cas9-mediated gene-editing system was used to generate *CsIAGLU* mutants in cucumber inbred line XTMC, a North China-type cucumber with a semi upright LPA. As shown in Figure 3A, two targets (Target 1 and Target 2) were designed at the first exon of *CsIAGLU*, and two homozygous mutants were obtained. The *Csiaglu#2* line had a 1 bp insertion at the second target, and the *Csiaglu#3* line had a 1 bp deletion at the second target (Figure 3A). Both mutant lines resulted in a truncated protein of CsIAGLU lacking the conserved UDPGT domain, with 280 amino acids in *Csiaglu#2* and 276 amino acids in *Csiaglu#3*.

A phenotypic analysis was conducted after 18-20 true leaves had been produced, and the average LPAs were calculated for the lower, middle and upper parts of the plant. Compared with the WT, the *Csiaglu* mutant lines had significant larger LPAs, especially the middle LPAs (Figure 3B,C). In the WT, the middle LPA was 42.8 ± 2.0°, while those of *Csiaglu#2* and *Csiaglu#3* were 53.8 ± 3.7°and 51.2 ± 1.3°, respectively (Figure 3C). The statistical analysis showed that there was a significant difference between the *Csiaglu* mutant lines and the WT. These results indicated that *CsIAGLU* is a negative regulator of LPA development in cucumber.

### 3.4. Csiaglu Mutant Displays Significant Enlargement of Adaxial Cell Size at Petiole Base

**Figure 4:**
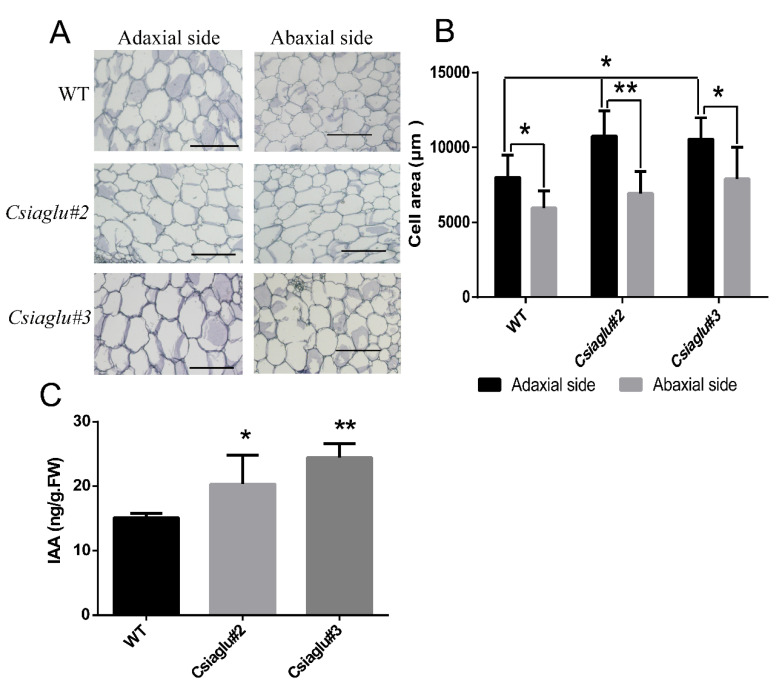
Cell size and auxin measurement in WT and *Csiaglu* mutant plants: (**A**) Cell morphology of transverse sections of petiole base in WT and *Csiaglu* mutants. Scale bar = 100 μm. (**B**) Cell area statistics in WT and *Csiaglu* mutants. (**C**) IAA contents at the petiole base. A significance analysis was conducted with the two-tailed Student’s *t*-test (* *p* < 0.05 and ** *p* < 0.01). Values are means ± SDs (*n* = 9 in (**B)**; *n* = 3 in (**C**)).

The cell size of the collar tissue determines the leaf angle in monocots [9,10,11]. A previous studies indicated that the free IAA of *OsGH3.1* gain-of-function mutant *lc1-D* was reduced and the adaxial cells were elongated, leading to greater leaf angle in rice [30]. Furthermore, *OsIAA6* (*Aux/IAA* gene) gain-of-function mutants displayed an enlarged leaf angle due to increased cell length on the adaxial side of the lamina joint [54]. To explore whether *CsIAGLU* regulates the LPA through cell expansion, the transverse section of the petiole base was compared between *Csiaglu* mutant and WT plants. Interestingly, the cell size on the adaxial side of *Csiaglu* mutant plants was significantly larger than that of the WT, while the cell size on the abaxial side was unchanged (Figure 4A,B). These data suggest that the enlarged LPA of *Csiaglu* mutants is due to increased cell expansion in the adaxial side of the pedicle in cucumber.

Previous studies show that both *ZmIAAGLU* and *OsIAAGLU* encode an IAA glucosyltransferase and that the level of free IAA in overexpression lines is significantly reduced [55,56,57]. To investigate whether *CsIAGLU* regulates the LPA through auxin homeostasis, we measured the content of IAA at the petiole base. As expected, the IAA level in *Csiaglu* mutants was significantly higher than in the WT (Figure 4C), indicating that the elevated LPA in *Csiaglu* mutants is due to auxin-mediated cell expansion on the adaxial side of petiole. 

### 3.5. IAA Treatment Results in Increased LPA in Cucumber

**Figure 5:**
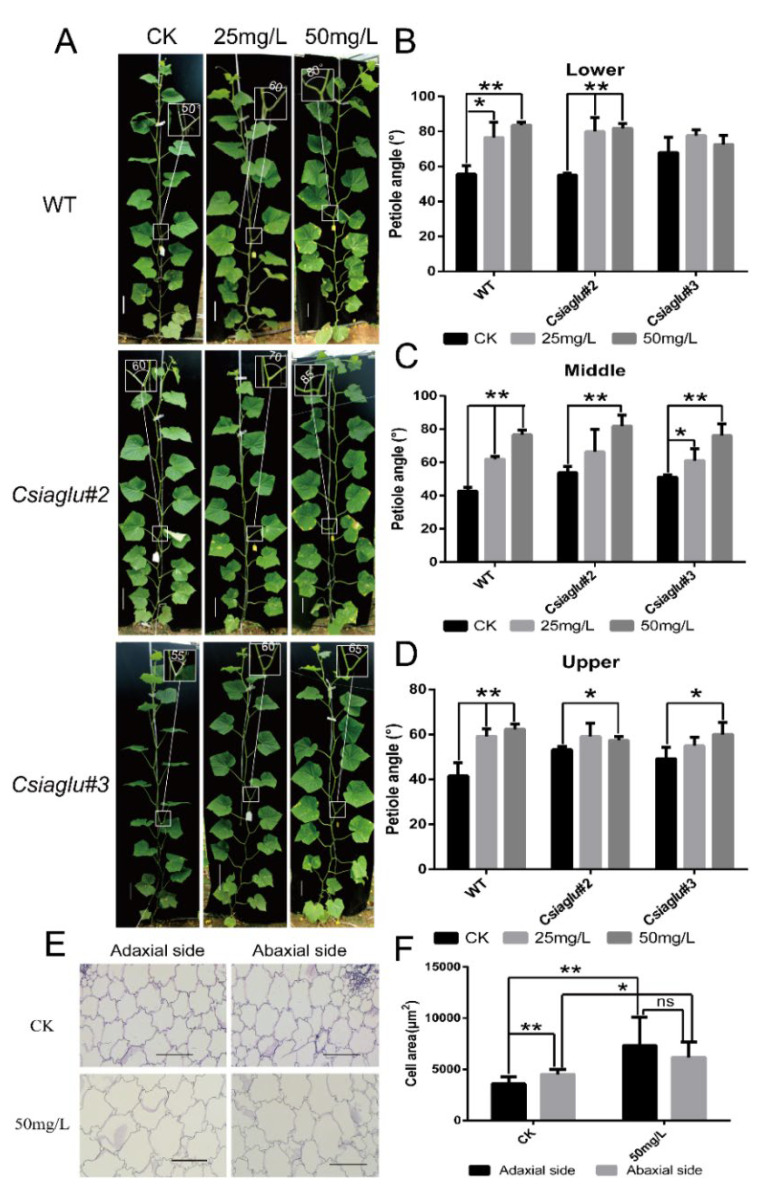
IAA treatment resulted in increased LPA in cucumber: (**A**) Representative images of WT and *Csiaglu* mutant plants following auxin application. The white boxes indicate the LPA of the largest leaf. Scale bars represent 10 cm. (**B**-**D**) Statistical analysis of LPAs of WT and *Csiaglu* mutants: lower LPAs (**B**), middle LPAs (**C**) and upper LPAs (**D**). Values are means of LPAs from the same part of three independent plants. (**E**) Cell morphology of transverse section of petiole base in CK plants and plants treated with 50 mg/L IAA. Scale bar = 100 μm. (**F**) Cell area statistics of petiole base in CK plants and plants treated with 50 mg/L IAA. A significance analysis was conducted with the two-tailed Student’s *t*-test (* *p* < 0.05 and ** *p* < 0.01). Values are means ± SDs (*n* = 3 in (**B**–**D**); *n* = 9 in (**F**)).

To verify the positive role of IAA in LPA development, exogenous IAA application was performed in the WT and *Csiaglu* mutants with different concentrations of IAA (25 mg/L and 50 mg/L). Compared with untreated (CK) plants, IAA application significantly increased the lower, middle and upper LPAs in both WT and *Csiaglu* mutant lines (Figure 5A–D), especially with 50 mg/L IAA treatment. The middle LPA increased 33.8° in WT, while those of *Csiaglu#2* and *Csiaglu#3* were 28.1° and 24.9°, respectively, following 50 mg/L IAA treatment (Figure 5C). These data are consistent with the positive role of IAA in LPA development in cucumber.

Next, we explored whether exogenous IAA treatment regulated the LPA by promoting cell enlargement. The cell size of the petiole base was measured in the WT following 50 mg/L IAA application. Our results showed that the cell sizes on the adaxial and abaxial sides of CK plants were 3586.2 ± 665.3 μm^2^ and 4519.3 ± 468.3 μm^2^, respectively, while those of treated plants were 7326.4 ± 2788.4 μm^2^ and 6182.4 ± 1487.1 μm^2^, respectively (Figure 5E,F). The quantification analysis indicated that IAA treatment significantly increased the cell sizes on both the adaxial and abaxial sides, with a greater increase on the adaxial side (2.04-fold) as compared to the abaxial side (1.36-fold). This result shows that auxin stimulates cell enlargement to increase the LPA in cucumber.

### 3.6. Auxin Transport and Auxin Signaling Are Unaffected in Csiaglu Mutants 

Previous studies show that auxin transporters, *PINs*, play important roles in regulating the leaf angle in maize, rice and soybean [33,58,59,60,61,62,63,64]. The expression of *CsPIN1a*, *CsPIN1b*, *CsPIN2*, *CsPIN4*, *CsPIN5* and *CsPIN8* was detected in *Csiaglu* mutants and WT plants. Our results indicated that there were no significant differences between *Csiaglu* and WT for all *CsPIN* genes (Figure S1A–F). Next, we detected the expression of auxin signaling pathway genes, including *CsLC3*, *CsLIP1*, *CsTIR1*, *CsARF1* and *CsARF6*, which were obtained through blast search of the homologous genes in rice [60]. Similarly, no changes were found between WT and *Csiaglu* mutants (Figure S1G–K). These data are consistent with the role of *CsIAGLU* in auxin homeostasis, instead of auxin transport or signaling pathway, in the regulation of the LPA in cucumber.

## 4. Discussion

In the 1970s, Mock et al. first proposed the ideal plant architecture of crops based on ten important parameters of maize, with the leaf angle being one of them [59]. Cucumber is cultivated throughout the world in open fields or in greenhouses, with the majority of production taking place in protected environments for fresh markets. Due to its advantages of effective light capturing and adaptation to high-density planting, the small LPA is one of the key components for ideal shoot architecture in greenhouse cucumber; thus, it serves as an important agronomic goal for cucumber breeding. Unlike in maize, rice and other cereal crops, the influencing factors of the cucumber LPA are much more complicated, mainly because of the vine stem. Therefore, the leaf petiole angle of cucumber is not only controlled by genetic factors but also regulated by the posture of the stem. To accurately assess the LPA, the main stem is required to hang upright timely during cultivation. 

Auxin plays essential roles in plant growth and development. Multiple studies have shown the important roles of auxin in the regulation of the leaf angle in crops [8,22]. In addition to free IAA, there are two alternative types of IAA conjugates: amide-linked IAA and ester-linked IAA. In thale cress, IAA is mainly stored in the form of amide conjugates, IAA-Aspartate (IAA-Asp) and IAA-Glutamate (IAA-Glu). However, in monocotyledon maize and rice, IAA conjugates are mainly stored as IAA ester conjugates [65,66]. Some enzymes catalyzing IAA to produce IAA conjugates have been found to participate in leaf angle regulation. For example, GH3 family members encode IAA amino synthetase, which can catalyze the binding of certain amino acids to free IAA. OsGH3-1, OsGH3-2 and OsGH3.13 were found to be involved in the regulation of leaf angle in rice [30,67,68]. IAA glycosylation occurs in all vascular plants, but its biological functions are largely unknown. The *ZmIAAGLU* gene was first cloned from maize grains; it encodes an IAA glucosyltransferase. ZmIAAGLU can catalyze IAA and UDPG (uridine-50-diphosphoglucose) to synthesize IAA-Glucose (IAA-Glc) during maize grain maturation [55]. The overexpression of *ZmIAAGLU* in thale cress results in shorter roots, curly leaves and insensitivity to IAA [55]. Thale cress *UGT84B1*, *UGT84B2*, *UGT75B1*, *UGT75B2*, *UGT74D1* and *UGT74E2* all encode IAA glucosyltransferase. Overexpression lines or dominant mutants of these genes lead to significantly reduced free IAA and abnormal plant development [69,70]. Rice *OsIAAGLU* is a homologous gene of *ZmIAAGLU*, and its expression is induced by exogenous IAA. The leaf angle of *OsIAAGLU* overexpression plants was reported to be dramatically increased [57]. However, there are no reports on the function of IAA glucosyltransferase in cucumber yet.

Here, we identified an IAA glucosyltransferase, CsIAGLU, in cucumber (Figure 1). We found two haplotypes of *CsIAGLU*, of which *CsIAGLU^717G^*^,*1234T*^ is the dominant allele, associated with a small LPA, whereas the *CsIAGLU^717C^*^,*1234A*^ allele is linked with a large LPA (Figure 1). *CsIAGLU* had high expression levels in leaves and petioles (Figure 2). In natural cucumber populations, the expression of *CsIAGLU* was negatively correlated with the LPA (Figure 2). The mutation of *CsIAGLU* resulted in elevated free IAA levels and enlarged cell size on the adaxial side of the petiole base, thus producing a greater LPA (Figure 3 and Figure 4). The exogenous application of IAA led to increased LPA and cell expansion (Figure 5). Therefore, *CsIAGLU* acts as a negative regulator of LPA development through auxin-mediated cell expansion in cucumber. 

Research results of *CsIAGLU* homologous genes show that *ZmIAGLU*, *OsIAAGLU*, *UGT84B1*, *UGT75B1* and *UGT75B2*, etc., all have IAA glucosyltransferase activity [55,57,69,70], so we speculate that *CsIAGLU* also has IAA glucosyltransferase activity. Although there is no direct evidence to prove that CsIAGLU has IAA glucosyltransferase activity, there is indirect evidence, such as the significant increase in the level of free IAA in *Csiaglu* mutants (Figure 4C) and the reduced sensitivity of *Csiaglu* mutants to exogenous IAA compared with the WT (Figure 5), supporting the view that CsIAGLU has enzyme activity. Through the analysis of 80 cucumber materials, we found that CsIAGLU has two haplotypes and is associated with the LPA. Interestingly, one of the haplotype mutations occurs in the UDPGT domain (Figure 1E), but whether this mutation affects the enzyme activity of CsIAGLU remains to be further studied.

**Figure 6:**
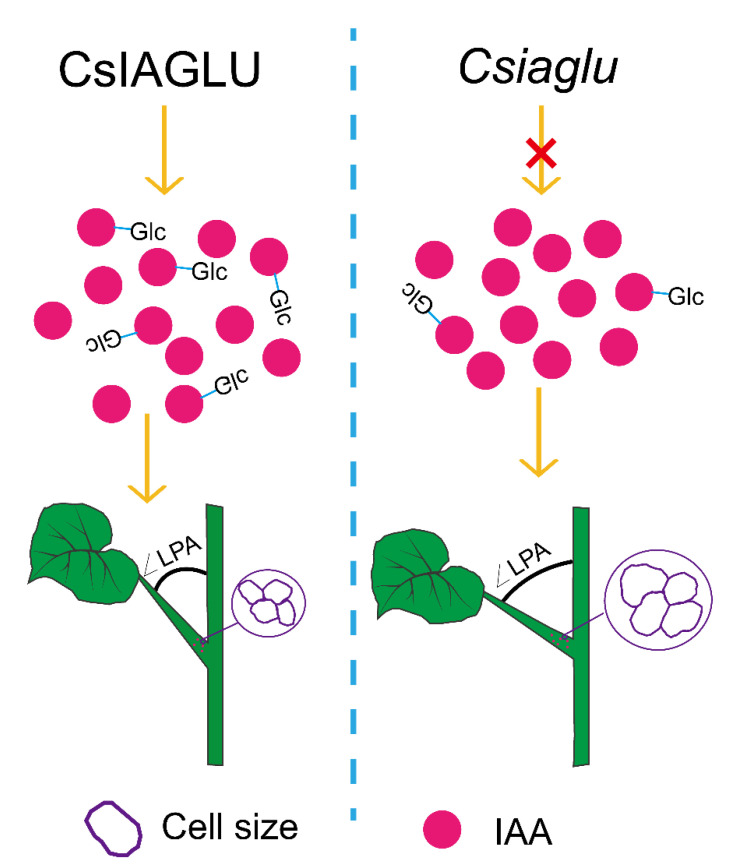
Proposed working model of *CsIAGLU* influence on cucumber LPA through regulating IAA concentration. CsIAGLU regulates endogenous IAA level by catalyzing free IAA to produce IAA-Glc. However, the increase in endogenous IAA concentration promotes the enlargement of adaxial cells at the petiole base, leading to a greater LPA.

In conclusion, our results show that in the WT, CsIAGLU catalyzes the glycosylation of free IAA to produce IAA-Glc; thus, it maintains appropriate free IAA levels and cell expansion at the base of the petiole for small-LPA development (Figure 6). In the *Csiaglu* mutant, IAA glucosyltransferase is perturbed, leading to elevated free IAA levels and increased cell expansion, thus generating large LPAs in cucumber (Figure 6). Interestingly, no developmental defects were observed in the *Csiaglu* mutant. Therefore, our study provides a valuable strategy for cucumber breeding with a small LPA, either by utilizing the *CsIAGLU^717G^*^,*1234T*^ allele or by elevating *CsIAGLU* expression, probably via CRISPR-Cas mediated gene editing in the promoter region. The expression analysis showed that auxin transport and auxin signaling pathway genes were unaffected in the *Csiaglu* mutant (Supplementary Figure S1). The downstream targets and underlying mechanism of *CsIAGLU* regulating the cucumber leaf petiole angle need further studies in the future. 

## Data Availability

The data used in this study are presented in the article or supplementary information.

## Supplementary Materials

The following supporting information can be downloaded at: https://www.mdpi.com/article/10.3390/genes13122216/s1, Figure S1: Expression analysis of IAA-response-related genes at petiole base of WT and Csiaglu mutants, Table S1: Gene information used in this study, Table S2: Primers used in this study, Supplementary Data S1: Correlation analysis of *CsIAGLU* SNPs and leaf petiole angle in cucumber germplasms.
