# Supplementary material for: CsIAGLU Regulates the Angle of Leaf Petiole by Affecting Endogenous Content of Auxin in Cucumber (Cucumis sativus L.)
Source: Genes (Basel). 2022 Nov 25;13(12):2216. doi: 10.3390/genes13122216 (PMC9777852; doi:10.3390/genes13122216)
Supplement: Supplementary file 1 [file genes-13-02216-s001.zip › genes-2031071-supplementary.pdf]

Table S1. Gene information used in this study.

Gene name	Species	Accession
AtIAGLU	*Arabidopsis thaliana*	AT4G15550.1
AtUGT74D1	*Arabidopsis thaliana*	AT2G31750.2
AtUGT74E2	*Arabidopsis thaliana*	AT1G05680.1
AtUGT75B1	*Arabidopsis thaliana*	AT1G05560.2
AtUGT75B2	*Arabidopsis thaliana*	AT1G05530.1
AtUGT84B1	*Arabidopsis thaliana*	AT2G23260.1
AtUGT84B2	*Arabidopsis thaliana*	AT2G23250.1
CsaV3_2G035510	*Cucumis sativus*	CsaV3_2G035510
CsIAGLU	*Cucumis sativus*	CsaV3_6G009300
CsaV3_6G032370	*Cucumis sativus*	CsaV3_6G032370
CsCRC	*Cucumis sativus*	CsaV3_5G033400
CsPIN1a	*Cucumis sativus*	CsaV3_1G007160
CsPIN1b	*Cucumis sativus*	CsaV3_1G004350
CsPIN2	*Cucumis sativus*	CsaV3_1G032010
CsPIN4	*Cucumis sativus*	CsaV3_5G013380
CsPIN5	*Cucumis sativus*	CsaV3_2G009610
CsPIN8	*Cucumis sativus*	CsaV3_3G041710
CsLC3	*Cucumis sativus*	CsaV3_3G046670
CsLIP1	*Cucumis sativus*	CsaV3_5G036540
CsTIR1	*Cucumis sativus*	CsaV3_7G028700
CsARF1	*Cucumis sativus*	CsaV3_1G000090
CsARF6	*Cucumis sativus*	CsaV3_1G036890
CsUBI	*Cucumis sativus*	CsaV3_5G031430
OsIAAGLU	*Oryza sativa Japonica Group*	XP_015630151.1
ZmIAAGLU	*Zea mays*	NP_001105326.1

Table S2. Primers used in this study.

**Primers for qRT-PCR**	
CsIAGLU-F	CGTCGTGGATCTGATGCTTTG
CsIAGLU-R	TACAATGCGAAAACGACAGCC
CsARF1-F	AACGAGCGCGTCTTCTACTT
CsARF1-R	GGAGGTGGAGGTTCCTTGTC
CsARF6-F	CGCTGGTCCTCTTGTTTCTCT
CsARF6-R	ACTCAAGGGTTGCAGAGTCA
CsLC3-F	TCAGAAGCTCCTAGAGGGGT
CsLC3-R	ACCATGTTTCCAGTCCCTCC
CsLIP1-F	GTCATGTCTGCCACAAGCAA
CsLIP1-R	TTCCTCTTCGGCATGAAACCT
CsPIN1a-F	TTGCTGCAGACACTCTCCAAA
CsPIN1a-R	AAGCTCCCTGAAAAATCCCCA
CsPIN1b-F	CGCCTCCAACAATCCCTACA
CsPIN1b-R	TAAGCTGCCAGAAAAATCGCC
CsPIN2-F	CTCCAACGATCCTTACGCCA
CsPIN2-R	TGGACCATAAGATTGCCAGAGA
CsPIN4-F	TTGCTCTTACGATTTGGGCG
CsPIN4-R	TCGCTCCACGATACTCAAACA
CsPIN5-F	CGTTCTCGTCTTGGCCTTTTG
CsPIN5-R	TTCAGCAGCGACCAAATCCA
CsPIN8-F	CTCGTGGAGGCTTGAATTGG

CsPIN8-R	GGAAGCTGCTTTGGTGGTAG
CsTIR1-q-F	CTGCTATGCCGTCAGTCCTC
CsTIR1-q-R	AGCTCCAAACTCTCATCCGT
CsUBI-F	CACCAAGCCCAAGAAGATC
CsUBI-R	TAAACCTAATCACCACCAGC
**Primers for in situ probes**	
CsIAGLU-sp6	GATTTAGGTGACACTATAGAATGCTTCATACCCCAACTCTCCCACA
CsIAGLU-T7	TGTAATACGACTCACTATAGGGTCACGAGCACTCAACAATGGA
**Primers for construction of CRISPR/Cas9 vector and mutants Identification**	
CsIAGLU-DT1-BsF	ATATATGGTCTCGATTGCTATCCGCCTACCGCCGTAGTT
CsIAGLU-DT1-F0	TGCTATCCGCCTACCGCCGTAGTTTTAGAGCTAGAAATAGC
CsIAGLU-DT2-R0	AACGCTCGTAGATCGAAATAGGCAATCTCTTAGTCGACTCTAC
CsIAGLU-DT2-BsR	ATTATTGGTCTCGAAACGCTCGTAGATCGAAATAGGCAA
CsIAGLU-clone-F	TTTAGTTACACATTGTGCTCAAGGC
CsIAGLU-clone-R	TAGACAACTGATGCTTTAGGTTTCG

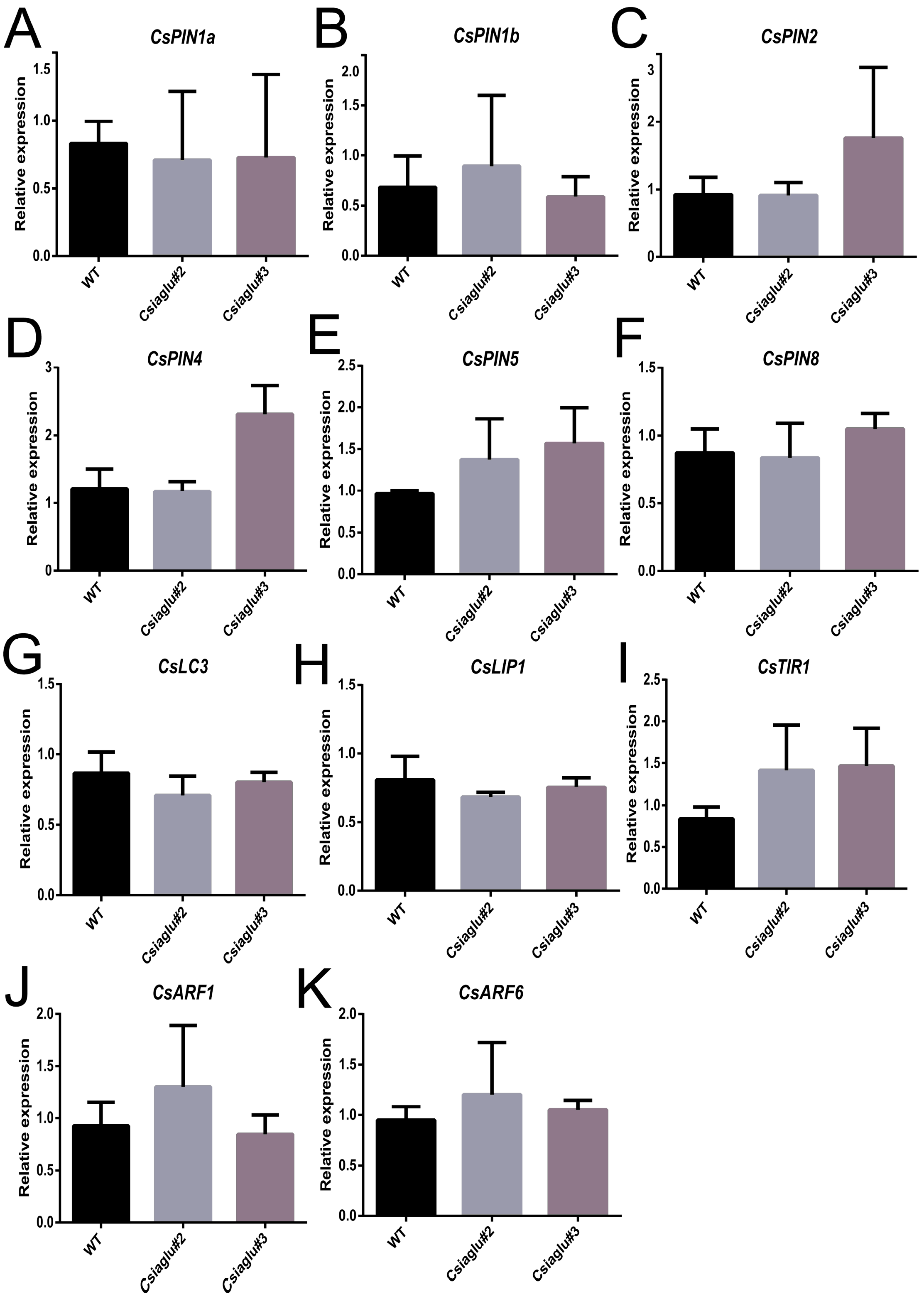

Supplementary Figure S1. Expression analysis of IAA response related genes in petiole base of WT and *Csiaglu* mutants. Significance analysis compared to WT was performed with the two-tailed Student's t test. Values are means ± SD (n = 3).
